# Clinical Acuity in the Emergency Department and Injury Severity Determine Hospital Admission of Older Patients with Low Energy Falls: Outcomes from a Prospective Feasibility Study

**DOI:** 10.3390/jcm12093144

**Published:** 2023-04-27

**Authors:** Valentin Clemens, Maximilian M. Saller, Rupert Meller, Carl Neuerburg, Christian Kammerlander, Wolfgang Boecker, Matthias Klein, Vera Pedersen

**Affiliations:** 1 Department of Orthopedics and Trauma Surgery, Musculoskeletal University Center Munich (MUM), University Hospital, Ludwig Maximilian University Munich (LMU), Marchioninistr. 15, 81377 Munich, Germany; valentin.clemens@med.uni-muenchen.de (V.C.); maximilian.saller@med.uni-muenchen.de (M.M.S.); carl.neuerburg@med.uni-muenchen.de (C.N.); wolfgang.boecker@med.uni-muenchen.de (W.B.); 2 Department of Orthopedics and Trauma Surgery, Klinikum Dritter Orden, Menzinger Str. 44, 80638 Munich, Germany; rupert.meller@dritter-orden.de; 3 Trauma Hospital Styria, Goestinger Straße 24, 8020 Graz, Austria; christian.kammerlander@auva.at; 4 Emergency Department, University Hospital, Ludwig Maximilian University Munich (LMU), Marchioninistr. 15, 81377 Munich, Germany; matthias.klein@med.uni-muenchen.de; 5 Department of Neurology, University Hospital, Ludwig Maximilian University Munich (LMU), Marchioninistr. 15, 81377 Munich, Germany

**Keywords:** older adult, low energy fall, hospital admission, tablet-based assessment, clinical frailty scale, emergency severity index, Charlson comorbidity index, injury severity

## Abstract

Background: Low energy falls (LEF) in older adults constitute a relevant cause for emergency department (ED) visits, hospital admission and in-hospital mortality. Patient-reported outcome measures containing information about patients’ medical, mental and social health problems might support disposition and therapy decisions. We investigated the value of a tablet-based (self-)assessment in predicting hospital admission and in-hospital mortality. Methods: Patients 65 years or older, consecutively presenting with LEF to our level I trauma center ED (from November 2020 to March 2021), were eligible for inclusion in this prospective observational study. The primary endpoint was hospital admission; secondary endpoints were in-hospital mortality and the use of the tablet for self-reported assessment. Multivariate logistic regression models were calculated to measure the association between clinical findings and endpoints. Results: Of 618 eligible patients, 201 patients were included. The median age was 82 years (62.7% women). The hospital admission rate was 45.3% (110/201), with an in-hospital mortality rate of 3.6% (4/110). Polypharmacy (odds ratio (OR): 8.48; 95% confidence interval (95%CI) 1.21–59.37, *p* = 0.03), lower emergency severity index (ESI) scores (OR: 0.33; 95%CI 0.17–0.64, *p* = 0.001) and increasing injury severity score (ISS) (OR: 1.54; 95%CI 1.32–1.79, *p* < 0.001) were associated with hospital admission. The Charlson comorbidity index (CCI) was significantly associated with in-hospital mortality (OR: 2.60; 95%CI: 1.17–5.81, *p* = 0.03). Increasing age (OR: 0.94; 95%CI: 0.89–0.99, *p* = 0.03) and frailty (OR: 0.71; 95%CI: 0.51–0.99, *p* = 0.04) were associated with the incapability of tablet use. Conclusions: The severity of fall-related injuries and the clinical acuity are easily accessible, relevant predictors for hospital admission. Tablet-based (self-)assessment may be feasible and acceptable during ED visits and might help facilitate comprehensive geriatric assessments during ED stay.

## 1. Introduction

Low-energy falls (LEF) occur in one-third of adults over the age of 65 each year and are a leading cause of death in developed nations [1]. The emergency department (ED) visit rates for LEF among older adults are increasing [2,3,4,5] with increasing odds for hospital admission [4,6,7] and significant morbidity and mortality that appear to increase with age [8,9,10]. Previous studies that exclusively focus on older adults with LEF in the ED report hospital admission rates between 40% [6] and 66% [11,12].

In light of increasing health care costs and resource utilization during ED visits and inpatient treatment, increasingly limited hospital beds and quality assurance and transparency of medical care, the disposition decisions in relation to older adults might not only require the assessment of medical issues but also of functional and social problems [13]. Therefore, an early and reliable prediction of hospital admission upon presentation to the ED of the older patient with LEF, reflecting both the acute fall-related injuries and the underlying acute and chronic health conditions, is required. This has been investigated by a few previous studies [6]. Besides validated instruments to measure frailty [14,15,16,17,18] or prognostic comorbidity [19,20], geriatric assessments, including patient-centered and reported outcome measures, might provide valid and valuable information on the quality of life, functionality, physical, mental and social health, and should directly support the disposition decision as well as surgical therapy [21,22,23,24]. Further, self-assessment of patient-reported outcome measures (PROMs), e.g., tablet-based, will help to provide transferable and interchangeable data for clinical utilization [25,26] and to improve the quality and patient-centeredness of acute care [27]. Large-scale evidencing studies showing the value of a tablet-based (self-)assessment of a certain panel of PROMs in predicting clinical outcomes of older patients in the ED with LEF are missing so far.

Therefore, the objective of this study was to investigate the value of a tablet-based (self-)assessment in older patients presenting to the ED with LEF in predicting hospital admission and in-hospital mortality and the feasibility of the technology in the acute ED setting.

## 2. Materials and Methods

The study is in accordance with the declaration of Helsinki and was conducted using STROBE guidelines. Ethical approval was obtained from the local ethic committee (EK LMU 19-177, approved 24 July 2019). Written informed consent was obtained from all participants prior to participation, and they were all assured that they could withdraw their consent at any time without consequence.

### 2.1. Study Design and Setting

This prospective observational single-center feasibility study was carried out in the emergency department (ED) of the University Hospital of Ludwig Maximilian University, Munich, an university tertiary care hospital. Data were acquired using a tablet-based survey combined with electronic health records (EHR). All study data were captured using the open-source RED-Cap (Research Electronic Data Capture, Vanderbilt University, Nashville, TN, USA) web-based survey platform.

### 2.2. Study Population

Potential patient participants were screened by two trained members of the study staff (V.C., V.P.) upon arrival to the ED for inclusion criteria between 1 November 2020 and 8 March 2021. The study population for recruiting included individuals ≥ 65 years of age, consecutively presenting to the ED and suffering from a LEF (as previously defined [12]: standing height, low-level furniture, low level/stairs ≤ 1 m or bi-cycle fall from standing position) in the previous 7 days. Exclusion criteria for an interview approach were an acute vital threat, not being fluent in the German language, diagnosed dementia or acute delirium resulting in an inability to consent, a presentation during the night-time hours (20:00 p.m.–8:00 a.m.) or non-acceptance of participation in the research study. Furthermore, patients with an acute infection or suspected acute COVID-19 disease were excluded.

### 2.3. Data Collection

From all recruitable patients consecutively presenting to the ED, the patients meeting the inclusion criteria for the interview approach were requested to give their informed written consent for participation by one of the two trained (V.C., V.P.) interviewers. After approval, the face-to-face tablet-based interview was conducted during the ED visit. First, every patient was asked to self-perform the PROMs survey using the tablet. Patients who declined or were unable to use the tablet or to perform the PROMs by self-assessment were questioned by the interviewers in a structured interview provided by the web-based RED-Cap survey platform. Patient-reported measures included internationally established instruments for measuring the quality of life, such as the Short Form Health 36 (SF 36) questionnaire [28] or the European Quality of Life 5 Dimensions 5 Levels (EQ-5D-5L) questionnaire [29], when the patient was not able to perform the SF-36 questionnaire due to cognitive impairments. Further, the Charlson Comorbidity Index (CCI) [19], known as a scoring instrument to quantify the comorbidity burden of 19 concomitant diseases; the Clinical Frailty Scale 9 (CFS-9) [30], validated to identify frailty in the ED [17]; the Barthel Index for assessing activities of daily living [31]; and the modified Confusion Assessment Method for the Emergency Department (mCAM-ED) validated instrument for screening of delirium in the ED [32] were recorded by the trained interviewers (V.C., V.P.) following the patients reported survey during the ED visit. Further baseline demographic data (age, gender, time and date of arrival, emergency severity index (ESI), disposition, length of ED stay (LOS ED), length of hospital stay, in-hospital mortality) and patients’ medical data (i.e., values from blood samples, current diagnosis, co-diagnosis, surgery performed, injury pattern and severity using Injury Severity Score (ISS) based on the Abbreviated Injury Scale (AIS) [33]) from the index visit and hospital stay where appropriate, were added (V.C.) to the RED-Cap database using patients’ EHR.

### 2.4. Key Outcome Measures

The primary endpoint of the study was hospital admission. Secondary endpoints were in-hospital mortality and tablet use for self-assessment of patient-reported measures.

### 2.5. Statistical Analysis

For descriptive statistics, the mean with standard deviation (SD) or median and range or interquartile ranges (IQRs) were used to report continuous and ordinal data, where applicable. The *T*-test was used to compare the central tendency for continuous data, and the Kruskal-Wallis test was used to compare the central tendency for ordinal data. Pearson’s Chi-squared test with Bonferroni correction for multiple comparisons was used for the comparison of categorical data. Logistic regression models were calculated to identify risk factors for the primary endpoint of hospital admission and the secondary endpoints in-hospital mortality and tablet use. *p* values < 0.05 were considered significant. IBM^®^ SPSS^®^ Statistics (V26.0) or R software v.4.1.2 (https://www.R-project.org/, accessed on 23 July 2022) and RStudio 2022.07.1 Build 554 were used to carry out the statistical analysis.

## 3. Results

**Figure 1:**
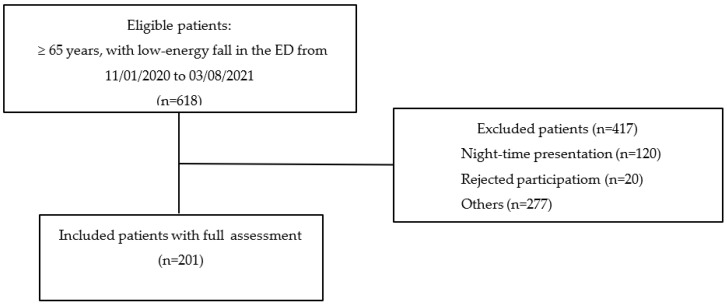
Inclusion and exclusion flow diagram of study patients from 1 November 2020 to 8 March 2021.

From 618 eligible patients, we included 201 patients in the tablet-based interview assessment and analysis (Figure 1). The median age was 82 years (IQR 78–88), and 126 of 201 (62.7%) of included patients were female. Table 1 gives an overview of baseline demographic information of the study cohort and the eligible patients. The hospital admission rate of the study patients was 45.3% (110/201), with an in-hospital mortality rate of 3.6% (4/110). Three of two hundred and one patients (1.5%) presented via the trauma bay. Supplementary Table S1 gives an overview of the demographic and clinical characteristics of study patients with interview-based assessment.

The mean LOS ED of the eligible patients was 5.38 h (SD: 2.6), while the mean LOS ED in the study group was 5.79 h (SD: 3.05) was not significantly different (see Table 1).

Table 2 summarizes the characteristics of the study group patients admitted to the hospital vs. patients who were discharged from the ED. Patients admitted to the hospital were more likely to take more than five medications (*p* = 0.009), to be more severely injured from the index fall (*p* < 0.001) and to be estimated as more urgent for treatment (ESI) upon arrival in the ED (*p* = 0.004). Binary multivariate logistic regression analysis identified polypharmacy (OR: 8.48; 95%CI 1.21–59.37, *p* = 0.03), lower ESI scores (OR: 0.33; 95%CI 0.17–0.64, *p* = 0.001) and increasing ISS (OR: 1.54; 95%CI 1.32–1.79, *p* < 0.001) as risk factors for hospital admission (Table 3). Overall, 19.9% of the study patients were injured severely (ISS ≥ 9) or very severely (ISS ≥ 16) due to the LEF. Anticoagulation medications, antiplatelet or anticoagulation, were not associated with hospital admission (Chi^2^: 0.05, *p* = 0.83) or mortality (Chi^2^: 0.65, *p* = 0.63). Analysis of laboratory parameters revealed hyponatremia in 34 of 187 (19.7%) patients with blood sampling on admission (summarized in Supplementary Table S2). Blood hemoglobin levels (OR: 0.81, 95%CI 0.67–0.98, *p* = 0.03) and serum creatinine levels (OR: 0.87, 95%CI 0.86–0.88, *p* < 0.001) were associated with hospital admission but not serum sodium levels (OR: 0.97, 95%CI 0.89–1.05, *p* = 0.42).

Binary logistic regression analysis showed a significant association between the CCI and in-hospital mortality in patients admitted to the hospital (OR: 2.60; 95%CI: 1.17–5.81, *p* = 0.03). No further risk factor of the analyzed data was associated with in-hospital mortality (see Supplementary Table S3).

Overall, 52 of the 201 patients approached (25.9%) were able to perform the tablet-based assessment in the ED without the assistance of the interviewers following short directions for use. Logistic regression analysis revealed a significant association between the capability to use the tablet with age (OR: 0.94; 95%CI: 0.89–0.99, *p* = 0.03) and the level of frailty CSF (OR: 0.71; 95%CI: 0.51–0.99, *p* = 0.042), and no significant association with the Barthel Index (OR: 1.03, 95%CI: 0.97–1.09, *p* = 0.35), the CCI (OR: 1.12; 95%CI: 0.93–1.35, *p* = 0.22), or the ISS (OR: 1.0, 95%CI: 0.91–1.1, *p* = 0.98). Capability for tablet use was not associated with hearing or visual impairment or the actual living condition.

## 4. Discussion

In this prospective observational study of a single-center university tertiary care hospital, we identified easily accessible clinical data that can help to identify elderly patients suffering from LEF with a high probability of being admitted. This is one of the first proof-of-principle studies that show that tablet-based assessed PROMs in elderly patients can be performed during the acute ED visit and reasonably support healthcare professionals in performing a thorough geriatric assessment in the ED.

Demographic changes and a growing geriatric population in the EDs [3,4,5,34] are associated with subsequently increased utilization of healthcare systems’ resources in the ED and even more when admitted to the hospital [35]. The increasing shortage of hospital beds and consideration of health expenses require proactive, easily accessible, reliable, valid and patient-centered criteria for disposition. Neither existing research on outcome prediction nor disease-specific tools [36] nor even a universal discharge score, as recently proposed [37], focus on older patients presenting to the ED due to LEF. Overall hospital admission rate in our study cohort was moderately high (45%) and within the range of admission rates of previous studies, which reported hospital admission rates ranging from 40% [6] up to 66% [11,12] in older patients with LEF. As previously shown, hospital admission in these patients is determined by the severity of fall-related injuries but also by fall dynamics and the individual’s clinical complexity, i.e., polypharmacy, anticoagulation intake or dementia [6]. In addition, our study demonstrates that besides a rather global surrogate for clinical complexity, i.e., polypharmacy, and the actual fall-related injury severity, the estimated acuity upon initial ED presentation, represented by the ESI level, is predictive of hospital admission. Although age seems to have an impact on hospital admission, as demonstrated in a large all-comer cohort of older adults presenting to the ED [7], age was not identified as a predictor for hospital admission in our study cohort. We assume that low-energy trauma reflects an acute health condition or an acute deterioration of chronic conditions, where acuity dominates the underlying conditions, including age as a predisposing factor. This assumption is supported by the finding that neither frailty, general health condition reflected by the CCI, nor the grade of performance in activities of daily living was associated with hospital admission in these patients with a LEF.

The ESI triage tool demonstrated validity when used to triage patients older than 65 years in the ED to predict hospital admission, length of hospital stay, resource utilization and survival [38,39]. However, it has been argued that the ESI score may not serve as the single best predictor for hospital admission, and age becomes a more significant predictor in old and very old patients [40]. The results of our study now add the valuable information that in older patients with LEF, acute fall-related injury severity is, besides ESI, a second strong predictor for hospital admission, taking low-energy trauma as a relevant health issue into account. Considering that fall-related complaints are one of the dominant reasons for ED presentation of older patients [2,3,4,5], this means for hospital resource planning that if at least every second patient with a simple fall is admitted for inpatient therapy, beds must be available for these patients, including surgical and intensive care capacity for those patients with severe and very severe injuries and requiring surgical interventions. With respect to the safety of this highly vulnerable patient population, this should be demanded from hospital management, politicians and healthcare systems.

We found blood hemoglobin and serum creatinine values associated with hospital admission but not in-hospital mortality. For both ESI and ISS, this might reflect the acuity of the patient’s condition, e.g., acute bleeding from the fall-related injury. In contrast to a previous study demonstrating a relevant association between hyponatremia and hospital admission due to a fall [41], serum sodium levels were not associated with hospital admission or in-hospital mortality in our observation. However, hyponatremia was prevalent in almost 20% of patients with initial laboratory work-up. This is higher than previously described for a general population of hospitalized geriatric patients (6.7%) [42], presumably underscoring that hyponatremia constitutes a relevant and preventable risk factor for injurious falls in older adults [43] and basic laboratory work-up in the ED should be mandatory in older patients with LEF.

The observed in-hospital mortality was 3.6% in our study cohort. This is comparable to a previously published large, retrospectively analyzed cohort of consecutive patients with LEF in our ED [12]. In a recent study, in-hospital mortality in patients with LEF was predicted only by the CCI. Previous studies demonstrated that the CCI, besides the Barthel Index, independently predicts hospital LOS, in-hospital mortality, and re-hospitalization in unselected non-surgical older patients admitted to the ED [20]. Although it had a low number of patients, our data supported the idea that acute trauma is significantly important for hospital admission, and once the older patient with LEF is hospitalized, the trauma severity no longer predicts mortality but rather general medical conditions and comorbidities regain prognostic importance.

The prevalence for frailty, as defined as a CSF-9 > 4 [30], in our study cohort was 21.4%. The clinical frailty scale CFS-9 was demonstrated to measure the general patients’ condition and frailty before ED presentation, even in older patients with major trauma [18,44]. The CSF-9 is highly reliable in measuring frailty in the ED [16,17] and is associated with adverse outcomes [14,45], and was therefore considered a useful instrument for decision-making with regard to triage, disposition and treatment [17]. However, in our LEF study cohort, frailty was not associated with hospital admission or in-hospital mortality, so it can be assumed that CFS-9 alone does not adequately identify older adults at risk for admission [15], in particular for these unique patients with low-energy trauma. In our study cohort, the majority of patients lived in nursing homes; therefore, the discharge decision might have been influenced by the agreement or disagreement of nursing home staff to monitor the patients in their domestic environment. Nevertheless, we advocate performing a valid assessment for frailty in the ED, on the one hand, for targeting specialist geriatric resources during hospital stay [46], on the other hand, to support triage or transfer to specialized trauma centers and surgical decision-making of the frail and very frail, as also suggested for older patients with major trauma [23,24].

This feasibility study demonstrates the proof of principle that a certain proportion of 25% of older patients in the ED is capable of performing tablet-based patient-reported measures and interviews in acute ED settings. This finding is supported by a few previous studies for the ED [26] and inpatient [25] setting with regard to older patients. The data provided by self-assessment on tablets were validated as reliable and amenable to widespread clinical utilization [25]. Limiting factors for independent tablet use in our investigation were age and frailty, which might reflect a general unskillfulness to use new technologies. However, this may change in the next 5 to 10 years as the generation of elders who are now familiar with new technologies come of age. No other factors defining the general health condition, sensory impairments, living conditions or acuity were limiting factors for tablet use. Furthermore, performing the full assessment with interviews did not result in a significantly prolonged LOS in the ED or a noticeable interference with standard ED work-up. Very recently, it has been emphasized that the successful adoption of PROMSs for diverse ED patient populations within the unique constraints of the acute care environment may help researchers, clinicians and policymakers improve the quality and patient-centeredness of acute care [27]. Our findings support the assumption that general requirements for more geriatric patient-centered awareness, and thereby early specific assessments of geriatric patients in the ED, are feasible and implementable and facilitated by means of modern standardizable and transferable web-based technologies.

Besides the strength of a thorough investigation of a highly relevant patient population in the EDs of high-income healthcare systems, this feasibility study has several limitations. Firstly, due to the monocentric design, the external validity with regard to the feasibility of the tablet-based assessment is limited. However, the study was designed as a proof-of-principle approach. In real-world terms, different ED work-up standards might limit patients’ access to interviewers or nursing practitioners. Furthermore, limited personal resources might limit the feasibility of a routine geriatric assessment 24/7, whether using institutional software or web-based open-source tools. Secondly, since the inclusion period was superimposed by the second wave peak of COVID-19 infections in Germany, there is a relevant risk of selection bias, thereby emphasizing patients’ selection of more frail and injured patients who were predominantly admitted by emergency medicine providers, where the less injured and compromised patients avoided to present to the ED. Thirdly, excluding patients with an acute vital threat (e.g., acute stroke) or without informed consent because of acute delirium or diagnosed dementia without authorized representatives constitutes another relevant risk for selection bias and underestimating the proportion of patients with relevant comorbidities, thereby underestimating the measured CCI, CSF and Barthel Index in the actual cohort. Finally, we should consider a volunteer bias, with a study population including patients that are more affine to new technologies and, therefore, more likely to participate in the study, thereby resulting in an overestimation of general feasibility.

## 5. Conclusions

This is a proof-of-principle study aiming to demonstrate the value of tablet-based (self-)assessed patient-reported measures in elderly patients with LEF to predict relevant clinical outcomes such as hospital admission and in-hospital mortality. Tablet-based self-assessments are feasible in a certain proportion of patients and acceptable during their ED visits. The acute fall-related injuries and clinical acuity upon initial presentation to the ED are relevant predictors for hospital admission, and the general health conditions of the patients are predictive of in-hospital mortality. To further support the assumption that digital-based technologies might facilitate geriatric assessment and patient-reported outcome measures in the ED with benefit for patients, health care providers and health care systems larger scale intervention trials are requested.

## Figures and Tables

**Table 1:** Baseline characteristics of 618 older adult patients presenting to the emergency department with low energy fell from 1 November 2020 to 8 March 2021, and the included study cohort with full assessment.

	Eligible Patients (*n* = 618)	Included Patients (*n* = 201)
Age (median, IQR)	83 (65–101)	82 (65–98)
Age 65–75 (%)	111 (20.4)	27 (14.4)
Age 75–85 (%)	260 (43.7)	97 (51.3)
Age > 85 (%)	247 (35.9)	77 (34.3)
Females (%)	392 (63.4)	126 (62.7)
Hospital admission (%)	359 (42.9)	110 (45.3) ^a^
Trauma bay presentation (%)	16 (2.6)	3 (1.5)
ED LOS all, median (hours) (95%CI)	4.83 (4.59–5.06)	5.17 (4.68–5.59)
Mean (hours) (SD)	5.38 (2.6)	5.79 (3.05) ^b^
ED LOS hospital-admitted	4.99 (4.49–5.31)	5.17 (4.60–5.92)
median (hours) (95%CI)		
Mean: 5.64 h (2.86)		
ED LOS discharged	4.67 (4.4–5.1)	4.8 (4.53–5.52) ^d^
median (hours) (95%CI)		
Mean: 5.34 h (2.58) ^c^		

The 95%CI = 95% confidence interval; ED LOS = length of stay emergency department; IQR = Interquartile range. ^a^ *p* < 0.05 Pearson’s Chi-squared test with Bonferroni correction for multiple comparisons, ^b^ *T*-test not significant between groups, ^c^ *T*-test not significant between dispositions, ^d^ ANCONA between groups not allowed due to heterogeneity.

**Table 2:** Demographic and clinical characteristics of patients of the study group (*n* = 201) with hospital admission compared to patients discharged from the emergency department.

	Study Patients (*n* = 201)	Hospital Admission (*n* = 110)	Discharged from ED (*n* = 91)	*p*-Value
Age (median, IQR)	82 (78–88)	82.5 (71.5–93.5)	81 (73–89)	0.28 ^a^
Females (%)	126 (62.7)	69 (62.7)	57 (62.6)	0.99 ^b^
Total Number of medications (mean, 95%CI)	5.5 (4.9–6.1)	5.76 (4.9–6.56)	5.15 (4.3–6.0)	0.41 ^a^
Polypharmacy (%)	100 (49.8)	60 (54.5) *	40 (44)	0.009 ^b^
CCI (Median, IQR)	5 (2–14)	5 (2–14)	5 (2–11)	0.167 ^a^
Barthel Index (mean, 95%CI)	92.7 (90.9–94.6)	92.9 (90.7–95.2)	92.5 (89.4–95.6)	0.31 ^a^
CFS-9 (Median, IQR)	3 (2.5–4)	3 (2–4)	3 (1–5)	0.42 ^a^
Vision impairment (%)	54 (26.9)	34 (30.9)	20 (22)	0.17 ^b^
Hearing impairment (%)	31 (15.4)	19 (17.3)	12 (13.2)	0.55 ^b^
ISS (median, IQR)	3 (2–4)	4 (0–18) **	1 (0–13)	<0.01 ^a^
ESI (median, IQR)	3 (1–4)	3 (1–4) **	3 (3–4)	0.004 ^a^
Mechanism of fall (%)				0.6 ^b^
Standing	159 (79.1)	89 (80.9)	70 (76.9)	
Furniture	19 (19.5)	11 (10)	8 (8.8)	
Below 1 m	14 (7)	8 (7.3)	6 (6.6)	
Bicycle	8 (4)	3 (2.7)	5 (5.5)	
unknown	1 (0.5)	1 (0.9)	0	
Level of care (%)				0.79 ^b^
Independent	23 (11.4)	12 (11.9)	11 (12.1)	
Nursing home	178 (88.6)	98 (89.1)	80 (87.9)	
Delay to presentation (%)				0.16 ^b^
0–24 h	172 (85.6)	96 (87.3)	76 (75.9)	
24–48 h	15 (7.5)	10 (9.1)	5 (5.5)	
48 h–7 days	12 (5.9)	3 (2.7)	9 (9.9)	
>7 days	2 (1)	1 (0.9)	1 (1.1)	

95%CI = 95% confidence interval; CCI = Charlson Comorbidity Index; CFS = clinical frailty scale; ESI = emergency severity index; IQR = interquartile range; ISS = injury severity scale; ^a^ Kruskal-Wallis test, ^b^ Pearson’s Chi-squared test with Bonferroni correction for multiple comparisons. The asterisk indicates different from discharged patients.

**Table 3:** Multivariate logistic regression analysis of potential risk factors for hospital admission (*n* = 201).

Variable	OR	95%CI	*p*-Value
Age	1.00	0.95–1.05	0.96
Polypharmacy	8.48	1.21–59.37	0.03
Barthel Index	1.02	0.99–1.06	0.24
CFS-9	1.19	0.86–1.63	0.29
CCI	1.15	0.96–1.39	0.14
ISS	1.54	1.32–1.79	<0.001
ESI	0.33	0.17–0.64	0.001

The 95%CI = 95% confidence interval; CCI = Charlson Comorbidity Index; CFS-9 = clinical frailty scale; ESI = emergency severity index; ISS = injury severity scale; OR = odds ratio.

## Data Availability

The datasets used and/or analyzed during the current study are available from the corresponding author upon reasonable request.

## Supplementary Materials

The following supporting information can be downloaded at: https://www.mdpi.com/article/10.3390/jcm12093144/s1, Table S1: Demographic and clinical characteristics of study cohort patients (*n* = 201); Table S2: Summary of laboratory parameters (mean, median, range) from initial blood sampling and proportion of pathological alterations; Table S3: Multivariate logistic regression analysis of potential risk factors for in-hospital mortality (*n* = 201); Figure S1: Representative screenshots of tablet-based surveys on RedCAP platform: (A) overview of surveys performed in this study; (B) extract of baseline data; (C) extract of SF-36 survey; (D) extract of assessed complaints; (E) assessment of fall history; (F) extract of EQ-5D-5L survey.
