# Supplementary material for: Clinical Acuity in the Emergency Department and Injury Severity Determine Hospital Admission of Older Patients with Low Energy Falls: Outcomes from a Prospective Feasibility Study
Source: J Clin Med. 2023 Apr 27;12(9):3144. doi: 10.3390/jcm12093144 (PMC10179013; doi:10.3390/jcm12093144)
Supplement: Supplementary file 1 [file jcm-12-03144-s001.zip › jcm-2298810-supplementary.pdf]

Table S1. Demographic and clinical characteristics of study cohort patients (n=201).

	**Study cohort (n=201)**
Delay to presentation	
0 to 24 hours (%)	172 (85.6)
24 to 48 hours (%)	15 (7.5)
48 hours to 7 days (%)	12 (12.6)
>7 days (%)	2 (1)
Readmission within 30 days (%)	11 (5.5)*
Emergency severity index (ESI)	
ESI 1	3 (1.5)
ESI 2	1 (0.5)
ESI 3	132 (65.7)
ESI 4	65 (32.3)
ESI 5	0 (0)
Mechanism of fall	
From standing height	159 (79.1)
From/out of furniture	19 (9.5)
Fall ≤1 meter	14 (7)
From bicycle while standing	8 (4)
unknown	1 (0.5)
Way of presentation	
Self-presentation	29 (14.4)
Referral by practitioner	9 (4.5)
Transfer from other hospital	5 (2.5)
Presentation by EMS	156 (77.7)
Presentation by EMS & emergency physician	2 (1)
Length of hospital stay (median, range)	7 (1-36)
Clinical frailty status (CFS-9) (median, range)	3 (2-7)
Moderate (1-4) (%)	158 (78.6)
Medium (5-6) (%)	32 (15.9)
Severe (7-9) (%)	11 (5.5)
Prevalence of delirium (mCAM ED) (%)	
Safe	5 (2.5)
Possible	1 (0.5)
none	195 (97)

Injury severity (ISS) (Median, 95%CI, range)	3 (2-4, 1-16)
Severe (ISS ≥ 9) (%)	36 (17.9)
Very severe (ISS ≥ 16) (%)	4 (2)
Non-injurious fall (ISS = 0)	5 (2.5)

95%CI = 95% confidence interval; EMS = emergency medical services; ESI = Emergency severity Index; ISS = injury severity scale; mCAM ED = modified Confusion Assessment Method for the ED *5 patients with repeated assessment.

Table S2. Summary of laboratory parameters (mean, median, range) from initial blood sampling and proportion of pathological alterations.

Variable	n	Mean (95%CI)	Median (95%CI)	Range	Pathological alterations (%)
blood hemoglobin (g/dl)	174	12.8 (12.5-13.1)	12.8 (12.6-13.4)	7.3-17.2	Anemia (≤ 13 g/dl) 89/174 (51.1)
serum sodium (mmol/l)	173	138.3 (137.7-138.9)	138.0 (138.0-139.0)	124-148	Hyponatremia (≤135 mmol/l) 34/173 (19.7)<br>Hypernatremia (≥ 146 mmol/l) 4/173 (2.3)
serum creatinine (mg/dl)	170	1.1 (0.9-1.2)	0.9 (0.9-1.0)	0.5-7.9	Creatinine ≥ 1.2 mg/dl: 37/170 (21.8)

95%CI = 95% confidence interval.

Table S3. Multivariate logistic regression analysis of potential risk factors for in-hospital mortality (n=201).

**Variable**	**OR**	**95%CI**	**p-value**
Age	1.01	0.83-1.22	0.95
Polypharmacy	0.13	0.003-6.43	0.30
Barthel Index	0.95	0.87-1.03	0.21
CFS 9	0.45	0.13-1.83	0.29
CCI	2.62	1.18-5.85	0.02
ISS	1.27	0.96-1.69	0.09
ESI	1.33	0.26-6.83	0.73

95%CI = 95% confidence interval; CCI = Charlson Comorbidity Index; CFS-9 = Clinical Frailty Index 9 Items; ESI = Emergency Severity Index; ISS = Injury Severity Scale.

A: Overview

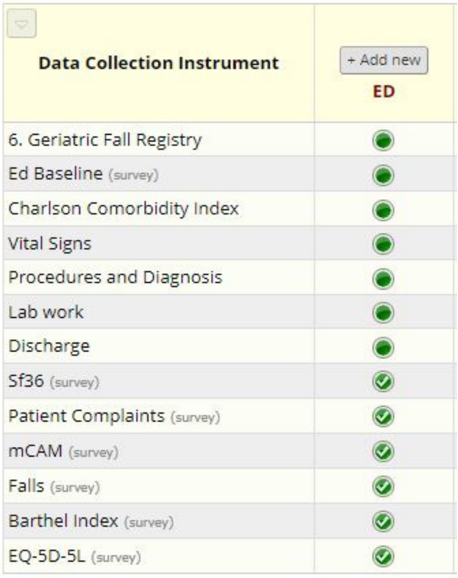

Data Collection Instrument	+ Add new ED
6. Geriatric Fall Registry	
Ed Baseline (survey)	
Charlson Comorbidity Index	
Vital Signs	
Procedures and Diagnosis	
Lab work	
Discharge	
Sf36 (survey)	
Patient Complaints (survey)	
mCAM (survey)	
Falls (survey)	
Barthel Index (survey)	
EQ-5D-5L (survey)	

B: ED Baseline

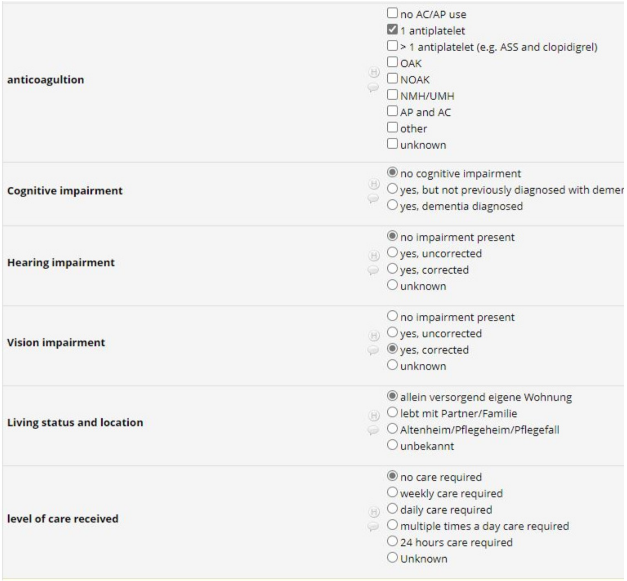

anticoagultion	no AC/AP use<br>1 antiplatelet<br>> 1 antiplatelet (e.g. ASS and clopidigrel)<br>OAK<br>NOAK<br>NMH/UMH<br>AP and AC<br>other<br>unknown
Cognitive impairment	no cognitive impairment<br>yes, but not previously diagnosed with demer<br>yes, dementia diagnosed
Hearing impairment	no impairment present<br>yes, uncorrected<br>yes, corrected<br>unknown
Vision impairment	no impairment present<br>yes, uncorrected<br>yes, corrected<br>unknown
Living status and location	allein versorgend eigene Wohnung<br>lebt mit Partner/Familie<br>Altenheim/Pflegeheim/Pflegefall<br>unbekannt
level of care received	no care required<br>weekly care required<br>daily care required<br>multiple times a day care required<br>24 hours care required<br>Unknown

C: SF36

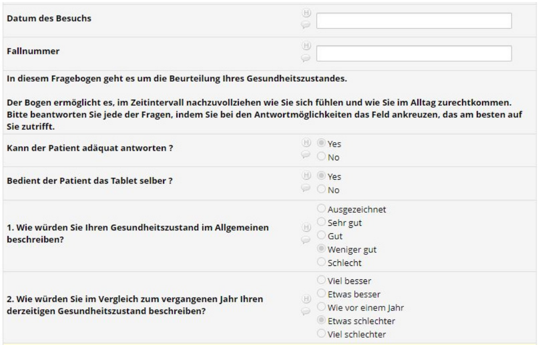

Datum des Besuchs	
Fallnummer	
In diesem Fragebogen geht es um die Beurteilung Ihres Gesundheitszustandes.<br>Der Bogen ermöglicht es, im Zeitintervall nachzuvollziehen wie Sie sich fühlen und wie Sie im Alltag zurechtkommen. Bitte beantworten Sie jede der Fragen, indem Sie bei den Antwortmöglichkeiten das Feld ankreuzen, das am besten auf Sie zutrifft.	
Kann der Patient adäquat antworten ?	Yes<br>No
Bedient der Patient das Tablet selber ?	Yes<br>No
1. Wie würden Sie Ihren Gesundheitszustand im Allgemeinen beschreiben?	Ausgezeichnet<br>Sehr gut<br>Gut<br>Weniger gut<br>Schlecht
2. Wie würden Sie im Vergleich zum vergangenen Jahr Ihren derzeitigen Gesundheitszustand beschreiben?	Viel besser<br>Etwas besser<br>Wie vor einem Jahr<br>Etwas schlechter<br>Viel schlechter

D: Patients Complaints

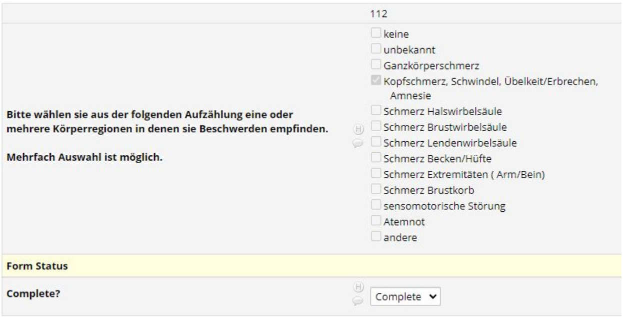

	112
Bitte wählen sie aus der folgenden Aufzählung eine oder mehrere Körperregionen in denen sie Beschwerden empfinden.<br>Mehrfach Auswahl ist möglich.	keine<br>unbekannt<br>Ganzkörperschmerz<br>Kopfschmerz, Schwindel, Übelkeit/Erbrechen, Amnesie<br>Schmerz Halswirbelsäule<br>Schmerz Brustwirbelsäule<br>Schmerz Lendenwirbelsäule<br>Schmerz Becken/Hüfte<br>Schmerz Extremitäten (Arm/Bein)<br>Schmerz Brustkorb<br>sensomotorische Störung<br>Atemnot<br>andere
Form Status	
Complete?	Complete

E: Falls

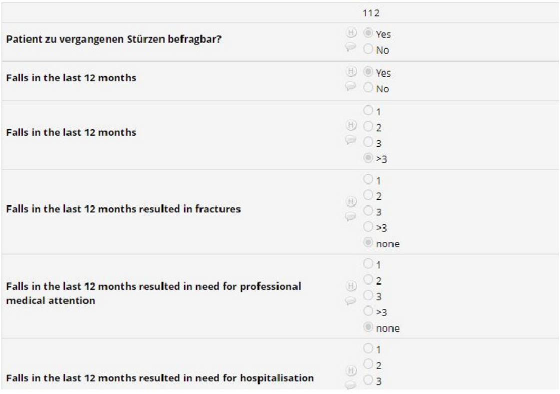

	112
Patient zu vergangenen Stürzen befragbar?	Yes<br>No
Falls in the last 12 months	Yes<br>No
Falls in the last 12 months	1<br>2<br>3<br>>3
Falls in the last 12 months resulted in fractures	1<br>2<br>3<br>>3<br>none
Falls in the last 12 months resulted in need for professional medical attention	1<br>2<br>3<br>>3<br>none
Falls in the last 12 months resulted in need for hospitalisation	1<br>2<br>3

F: EQ-D5-5L

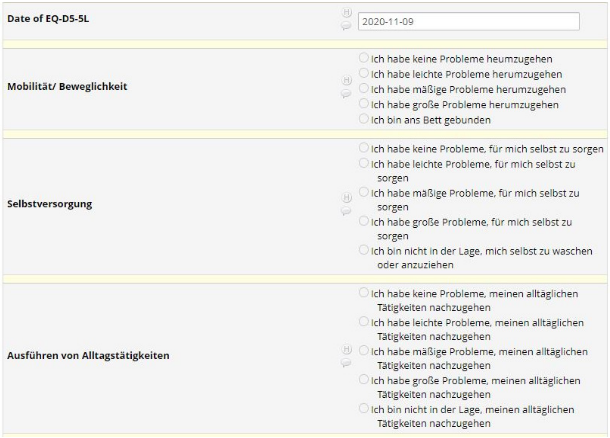

Date of EQ-D5-5L	2020-11-09
Mobilität/ Beweglichkeit	Ich habe keine Probleme heumzugehen<br>Ich habe leichte Probleme herumzugehen<br>Ich habe mäßige Probleme herumzugehen<br>Ich habe große Probleme herumzugehen<br>Ich bin ans Bett gebunden
Selbstversorgung	Ich habe keine Probleme, für mich selbst zu sorgen<br>Ich habe leichte Probleme, für mich selbst zu sorgen<br>Ich habe mäßige Probleme, für mich selbst zu sorgen<br>Ich habe große Probleme, für mich selbst zu sorgen<br>Ich bin nicht in der Lage, mich selbst zu waschen oder anzuziehen
Ausführen von Alltagstätigkeiten	Ich habe keine Probleme, meinen alltäglichen Tätigkeiten nachzugehen<br>Ich habe leichte Probleme, meinen alltäglichen Tätigkeiten nachzugehen<br>Ich habe mäßige Probleme, meinen alltäglichen Tätigkeiten nachzugehen<br>Ich habe große Probleme, meinen alltäglichen Tätigkeiten nachzugehen<br>Ich bin nicht in der Lage, meinen alltäglichen Tätigkeiten nachzugehen

Figure S1: Representative screenshots of tablet-based surveys on RedCAP platform: (A) overview of surveys performed in this study; (B) extract of baseline data; (C) extract of SF-36 survey; (D) extract of assessed complaints; (E) assessment of fall history; (F) extract of EQ-5D-5L survey.
